# Fall Risk Assessment Scales: A Systematic Literature Review

**DOI:** 10.3390/nursrep11020041

**Published:** 2021-06-02

**Authors:** Veronica Strini, Roberta Schiavolin, Angela Prendin

**Affiliations:** 1 Clinical Research Unit, University-Hospital of Padua, 35128 Padua, Italy; veronicastrini@gmail.com; 2 Continuity of Care Service-University-Hospital of Padua, 35128 Padua, Italy; roberta.schiavolin@aopd.veneto.it; 3 Independent Research, University-Hospital of Padua, 35128 Padua, Italy

**Keywords:** fall, scale, assessment tool, review

## Abstract

Background: Falls are recognized globally as a major public health problem. Although the elderly are the most affected population, it should be noted that the pediatric population is also very susceptible to the risk of falling. The fall risk approach is the assessment tool. There are different types of tools used in both clinical and territorial settings. Material and methods: In the month of January 2021, a literature search was undertaken of MEDLINE, CINHAL and The Cochrane Database, adopting as limits: last 10 years, abstract available, and English and Italian language. The search terms used were “Accidental Falls” AND “Risk Assessment” and “Fall Risk Assessment Tool” or “Fall Risk Assessment Tools”. Results: From the 115 selected articles, 38 different fall risk assessment tools were identified, divided into two groups: the first with the main tools present in the literature, and the second represented by tools of some specific areas, of lesser use and with less supporting literature. Most of these articles are prospective cohort or cross-sectional studies. All articles focus on presenting, creating or validating fall risk assessment tools. Conclusion: Due to the multidimensional nature of falling risk, there is no “ideal” tool that can be used in any context or that performs a perfect risk assessment. For this reason, a simultaneous application of multiple tools is recommended, and a direct and in-depth analysis by the healthcare professional is essential.

## 1. Introduction

The phenomenon of falls is recognized globally as a major public health problem. Falling down is globally the number-one health problem, and a common problem of evaluation by healthcare professionals. A fall is defined as a “sudden, not intentional, and unexpected movement from orthostatic position, from seat to position, or from clinical position” [1]. Falls involve elderly people for two main reasons: (1) the decrease of functional reserves that are used to maintain the orthostatic position; (2) the following vulnerabilities or pathologies caused by factors that occur simultaneously, pathological processes, and adverse pharmacological incentives. People over 65 have the highest probability of falling down: 30% of them fall down at least once per year, while the percentages become higher, (about 50%) on people over 80 [2]. Even if elderly people run the highest risk of falling down, it is necessary to point out that the pediatric population runs quite a high risk of downfall as well. About three million children are victims of wounds related to annual falls [3]. Although nearly 40% of the total daily falls worldwide occur in children, this measurement may not accurately reflect the impact of fall-related disabilities for older individuals who have more disabling outcomes and are at greater risk of institutionalization [1].

The financial costs of fall injuries are substantial. For people aged 65 and over in Finland and Australia, it was calculated at USD 3611 and USD 1049, respectively. Evidence from Canada suggests implementing effective prevention strategies with a subsequent 20% reduction of the incidence of falls among children under 10 could create net savings of more than USD 120 million annually [1]. The Joint Commission International for Accreditation Standards for Hospitals specifies that hospitals should aim to reduce the risk of injury from falls to inpatients and outpatients, including appropriate screening or assessment of fall risk tools, a process for re-evaluation, especially if there are changes in the patient’s condition; and implement interventions to reduce the risk of falling [4]. For this reason, risk assessment is important. The expression of risk assessment is based on the following: checklists drafted of different risk factors for fall and numerical indexes to predict the risk. The checklists help the staff to identify the most common factors, while the numerical index is used to predict the risk of an individual using a numerical score that is proportional to the number of risk factors included [5].

However, the characteristics of the patient for a fall risk tool are varied: age, cognitive state, state of health in general, particular comorbidities, hospital or home context. These are just a few features. In fact, in recent studies, particular risk factors have been evaluated such as being hospitalized, being hospitalized in neuropsychiatry, suffering from dementia and delirium, and going to the bathroom [6].

Currently in the literature, there is no study that summarizes the tools available to healthcare professionals according to the different contexts in which they operate. Knowing the fall assessment tools, through the analysis of their characteristics, allows to identify the most suitable scale for each individual patient and to prevent the risk. As described in NICE, 2004, the patients should be cared for by personnel who have undergone appropriate training and who know how to initiate and maintain correct and suitable preventative measures. Staffing levels and skill mix should reflect the needs of patients [6].

This review aims to analyze different fall risk assessment tools present in the literature with the aim of supporting healthcare professionals in choosing the tool best suited to their operational context and the characteristics of the patients.

## 2. Material and Methods

A literature search was undertaken from MEDLINE, CINHAL and The Cochrane Database in the month of January 2021.

The present review of literature followed the PRISMA guidelines [7]. The PICO method was adopted [8] as shown below to find the correct research terms to use:

Population: individuals who are in hospital environments or who stay in the territory, without any age limit.
Intervention: application of instruments to evaluate the risk of falling.
Comparison: none.
Outcomes: measurement of the downfall risk.

For every database, the following search terms were used: “Accidental Falls” and “Risk Assessment”, “Fall Risk Assessment Tool” or “Fall Risk Assessment Tools”.

The limits applied to each study were: year of publication 2010–2020 (last ten years); abstract available, Italian and English languages. Specific criteria of inclusion and exclusion were defined as shown below:

***Inclusion Criteria:*** Revision and research studies (experimental, observational and descriptive studies) focused on the presentation, creation, validation, or critique of instruments of fall risk evaluation.

***Exclusion criteria:*** articles that report evaluations measuring the risk of falling with medical parameters or criteria (ex. Laboratory data), or evaluations made through movement sensors; articles without abstract or not available in Italian or English languages.

**Figure 1:**
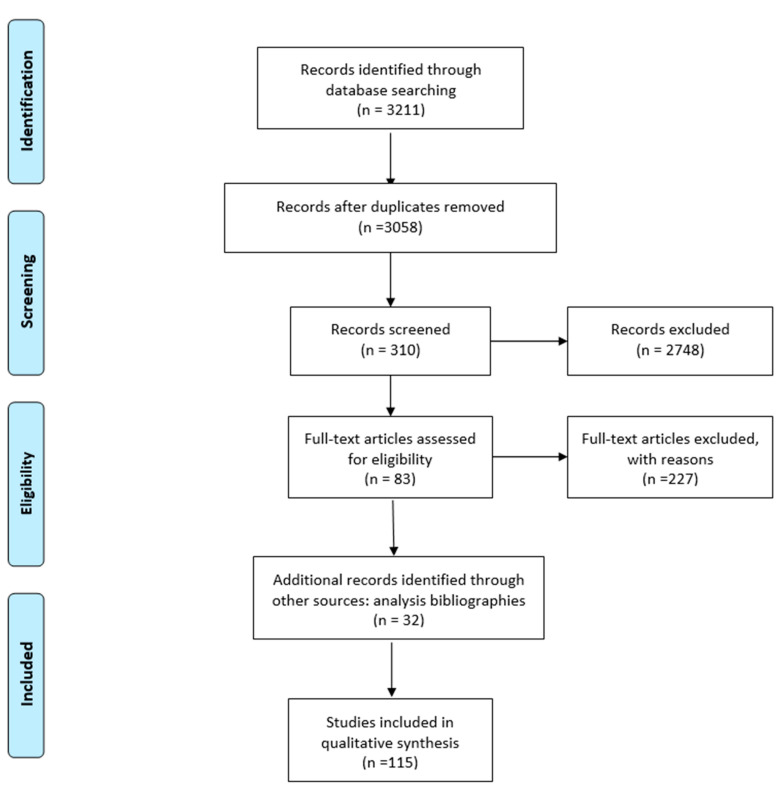
PRISMA flow diagram.

The review was not registered in any database or similar, and it was conducted by two separate reviewers who then compared the results on the basis of the limits and eligibility criteria chosen. The agreement was found as shown in Figure 1.

## 3. Results

Figure 1 is an illustrative example of the selection process with the final total number of articles included (n = 115). Most selected studies are prospective cohort studies or transverse studies, focused on the validation of an instrument in a different environment from the original one. In the other articles: retrospective studies, descriptive studies, methodical studies, and systematic reviews are present. Some instruments of evaluation have been compared to analyze their properties in relation to each other.

For the selected scales, the history and any important changes over the years have been reconstructed. Each scale, in fact, belongs to different populations or contexts, for the purpose of presenting the entire scenario of the tolls for assessing the risk of falling.

Tools identified by the analysis of the articles are 38, divided into two groups.

The first group is represented by the main 21 risk assessment tools, described in Table 1.

**Table 1:** Risk of falling evaluation tools.

Scale, Reference, Country	Rate	Language—Year of Validation	Sample	Time of Administration	How to Use
Tinetti Performance-Oriented Mobility Assessment [9] USA.	Score 0–28. <18–19 patient at risk of falling	English 1986; German 2017; Korean 2018.	Hospital setting (Parkinson’s disease, patients with amyotrophic lateral sclerosis, Huntington’s disease and community-resident elderly).	5 to 10 min	Performance.
Morse Fall Scale [10] Canada.	Score 0–125. 0–20 No risk or low risk; ≥25 Medium risk; ≥45, 50–55 High risk.	English 1989; German 2006; Chinese 2007; Korean 2011 Portuguese and Brazilian 2013.	Hospital setting (acute patient, rehabilitation and nursing home departments).	2 min.	Self-report.
Timed Up and Go (TUG) test [11] Canada.	Risk of falling if test time is >13.5 s. Most used cut-off in the literature.	English 1991: Brazilian 2012; Chinese 2017.	Hospital setting + screening of population (acute patients or community residents, individuals with different health alterations such as Parkinson’s syndrome or mental disabilities).	1 to 3 min.	Performance.
Berg Balance Scale (BBS) [12] Canada.	Score 0–56. <45 patient at risk of falling.	English 1992; Norwegian 2007; Brazilian 2009; Arabic 2016.	Hospital setting + screening of population (elderly living in communities or suffering from chronic diseases or with intellectual and visual disabilities, neuromuscular pathologies).	20 to 30 min.	Performance.
Downton Fall Risk Index [13] England.	Score 0–11. ≥3 patient at risk of falling.	English 1993; Spanish 2015; German 2003.	Hospital setting (post-stroke rehabilitation).	N/a.	Self-report.
Activities-specific Balance Confidence Scale (ABC Scale) [14] Canada.	Percentage value attributed of 0–100%. <50 Low level of functionality; 50–80 Medium level of functionality; >80 High level of functionality.	English 1995; Swedish 2003 Chinese 2006; French 2006; Portuguese 2013; Arabic2016.	Screening of population (elderly living home, people with Parkinson’s Syndrome, post-stroke, lower limb amputations and vestibular disorders).	20 min or less.	Self-report.
Dynamic Gait Index (DGI) [15] USA.	Total score 0–24. <19 at risk of falling.	English 1997.	Hospital setting + screening of population (elderly people, subjects suffering from vestibular dysfunction, multiple sclerosis and post-stroke).	15 min.	Performance.
St. Thomas Risk Assessment Tool in Falling Elderly Inpatients (STRATIFY) [16] England.	Score 0–5. ≥2 patient at risk of falling.	English 1997; Italian 2014; Spanish 2017.	Hospital setting (ICU, geriatric and rehabilitation departments).	3 min.	Self-report.
Conley Scale [17] USA.	Score 0–10. 0–2 no risk; ≥2 patient at risk of falling; ≥8 high risk.	English 1999; Italian 2002.	Hospital setting (medicine and surgery departments).	2 min.	Self-report.
Minimal Chair Height Standing Ability Test (MCHSAT) [18] Australia.	Performance > 47 cm = Very high risk; performance 34–47 cm = High risk; performance < 34 cm Low risk.	English 2002.	Hospital setting + screening of population (heart disease or stroke).	N/a.	Performance.
Aachen Falls Prevention Scale [19] Germany.	Score 0–10. ≤5 High risk of falling.	German 2004.	Screening of population (no specific population, home care context).	N/a.	Self-report + Performance.
Falls Risk for Older Persons-Community Setting Screening Tool (FROP Com Screen) [20] Australia.	Score 0–45. 0–5 Low risk; 6–20 Medium risk; 21–45 High risk.	English 2004. Chinese and Thai 2017.	Hospital setting (subacute patients’ departments).	N/a.	Self-report.
Five Times Sit to Stand Test (5T-STS) [21] USA.	Time taken ≥15 s = at risk of falling.	English 2005.	Hospital setting + screening of population (Parkinson’s syndrome, stroke, arthritis of the lower limbs).	N/a.	Performance.
Falls Efficacy Scale—International (FES-I) [22] England.	Score 16–64.	English 2005; Brazilian 2010; Portuguese 2011; Turkish 2012; Persian 2013.	Screening of population (no specific context, home care ederly).	N/a.	Self-report.
Johns Hopkins Fall Risk Assessment Tool (JHFRAT) [23] USA.	Score 0–35. 0–6 Low risk; 7–13 Medium risk; 14–35 High risk.	English 2005. Chinese 2016; Brazilian 2016; Korean 2011; Persian 2018.	Hospital setting + screening of population (ICU, medicine departments).	5 min.	Self-report.
Fullerton Advanced Balance (FAB) Scale. [24] USA.	Score 0–40.	English 2006; German 2011.	Screening of population (functionally independent seniors).	10 to 12 min.	Performance.
Hendrich II Fall Risk Model [25] USA.	Score 0–16. ≥5 patient at risk of falling.	English 2007; Italian 2011; Portuguese 2013; Lebanese nel 2014; Chinese 2011.	Hospital setting (adult patients at risk in acute care hospitals).	10 min or less.	Self-report + Performance.
Medication fall risk score [26] USA.	Score: 3 points for each drug of the first item, 2 for each of the second item, 1 for the drug of the third one. ≥6 a Risk of falling.	English 2009.	Hospital setting (pharmacist-coordinated falls prevention program, patients with high risk drug therapy).	N/a.	Self-report.
Mini Balance Evaluation Systems Test (Mini-BESTest) [27] Italy.	Score 0–28.	Italian 2009.	Hospital setting (Parkinson’s syndrome).	10 to 20 min.	Performance.
Stopping Elderly Accidents, Deaths, and Injuries (STEADI) [28] USA.	Answer no to all questions = Low risk; at least one answer yes to the questions and passing the tests (hold the position for >10 s in each phase and get up from the chair more than 5 times in 30 s or less) = Medium risk; failure to pass the tests or report numerous falls or with hip fracture = High risk.	English 2013.	Hospital setting + screening of population (routine practice).	N/a.	Self-report + Performance.
Austin Health Falls Risk Screening Tool (AHFRST) [29] Australia.	Answer “Yes” to one of the items = at Risk of falling. Answer “No” to each item = Not at risk.	English 2017.	Hospital setting (acute and subacute patients’ departments).	N/a.	Self-report.

Table 2 describes the second group, with 17 additional assessment tools specific to some areas, but of lesser use and with less supporting literature. The tools divided by scope are the following: psychiatric field: “Baptist Health High Risk Falls Assessment (BHHRFA)”, “Wilson-Sims Fall Risk Assessment Tool (WSFRAT)”; pediatric field: “4-item Little Schmidy Pediatric Hospital Fall Risk Assessment Index”, “Humpty Dumpty Fall Scale (HDS)”, “Bayındır Hospital Risk Evaluation Scale for In-hospital Falls of Newborn Infants”; emergency department: “KINDER 1 Fall Risk Assessment Tool”, “Memorial Emergency Department (MED-FRAT)”; rehabilitation field: “Casa Colina Fall Risk Assessment Scale (CCFRAS)”, “Predict_FIRST”, “Marianjoy Fall Risk Assessment Tool (MFRAT)”, scope of home care: “Simple clinical scale”, “Home Falls and Accidents Screening Tool (HOME FAST)”; patients affected by stroke: “Stroke Assessment of Fall Risk (SAFR)”, “Royal Melbourne Hospital Falls Risk Assessment Tool (RMH FRAT)”, “Sydney Fall Risk Screening Tool”, “Outdoor Falls Questionnaire”, “Questionnaire for Fall Risk Assessment in the Elderly”.

## 4. Discussion

The vast majority of the proposed tools have been developed for use in acute and geriatric settings, in which there are numerous factors that expose individuals to this risk. The target most subjected to the assessment is the elderly population (>65 years), followed by people suffering from pathologies that alter walking and balance skills (e.g., Parkinson’s disease, mental disabilities, stroke outcomes, etc.).

Falls Efficacy Scale—International (FES-I). The FES-I scale is the most used tool in literature for the “fear of falling” evaluation, a factor closely related to the genesis of falls [22]. This scale refers only to basic daily activities of frail elderly people or people with disabilities. The FES-I includes 16 daily life activities, and the individual must report the perceived degree of concern in implementing each of the activities listed. It is the ideal tool for investigating the “fear of falling” of the elderly in normal daily activities.

The Activities-specific Balance Confidence Scale (ABC Scale) was developed to assess the perceived degree of confidence in maintaining balance or not becoming unstable in performing various functional tasks. It is a structured questionnaire that measures the confidence of an individual in carrying out activities and consists in attributing a percentage value, between “insecurity” and “complete security”, to the 16 proposed activities [14]. The ABC scale is simple to complete, and the time required for filling out can be as much as 20 min, which is why a simplified version has been proposed that includes six of the most challenging activities of the previous scale. The scale has been validated for use with different ratings including people with Parkinson’s Syndrome, post-stroke, with lower limb amputations and vestibular disorders; it has also been translated into several languages besides English such as Swedish, Chinese, Canadian French and Arabic [47].

Comparing the two scales, it emerged that the FES-I scale has a greater appropriateness of use in clinical settings than the ABC Scale, whose use is recommended mainly in the elderly living at home [22].

The STRATIFY scale is a predictive tool for the risk of falls in hospitalized patients. The compilation of the scale is not performed through direct observation of the patient, but the evaluator reports the score based on information obtained from the previous observation or from other caregivers. STRATIFY has been extensively studied in intensive care units in Australia, Europe and Canada [48] and has also been applied in numerous geriatric and rehabilitation departments [49]. In these contexts, it has long been considered the “Gold standard” tool to be used at patient admission thanks to the high sensitivity value demonstrated by numerous studies (between 73.7% and 93.0%) and the simplicity and speed of application (3 min).

At the same time, some studies criticize its reduced specificity, or identify its scarce usefulness if applied in different or specific contexts such as rehabilitation from traumatic brain injury, or in patients younger than 65 years old. The Hendrich II Fall Risk Model (HIIFRM) was designed to identify adult patients at risk of falling in acute care hospitals [25]. Unlike the STRATIFY scale, the history of previous falls was not considered as a risk factor. They are also taken into account due to drug categories that are at greater risk for falls and side effects than other drug categories. The time required for its compilation is approximately similar to that of the STRATIFY scale. The scale was tested on acute phase patients with different diagnoses (diabetes mellitus, stroke, heart failure), demonstrating that its effectiveness varies according to the patient group, the healthcare professional’s skill level and the clinical units in which is applied.

The Johns Hopkins Fall Risk Assessment Tool (JHFRAT) was used for the multi-factorial assessment of the risk of falling in departments for acute patients [23]. The JHFRAT scale is a tool that makes possible to implement a multi-factorial assessment of the risk of falling in a simple way, which requires an average of 5 min for its completion and is widely used in adult departments in the acute phase [50]. However, there are discordant results in the literature regarding its statistical characteristics: in some studies, a high sensitivity is reported, but a low specificity, vice versa in other studies, which consider it weak if used in specific contexts, such as in the departments of medicine [50,51].

The Tinetti Mobility Test (TMT) or the Performance-Oriented Mobility Assessment (POMA) consists of the combined use of the useful components drawn from both approaches [9]. The total POMA (POMA-T) consists of two sub-scales: the balance rating scale (“balance scale” or POMA-B) and the gait rating scale (“gait scale” or POMA-G) [52]. The scale is also used in different clinical contexts: its effectiveness was analyzed on patients with Parkinson’s disease, with amyotrophic lateral sclerosis, Huntington’s disease and community-resident elderly [52]. The time taken is 5–10 min, but it requires training for the examiner as a prerequisite and requires some equipment (stopwatch, chair, 5-pound object = about 2.5 kg and a space to walk 15 feet = about 5 m). It can also be burdensome for patients. There is a “long” version of the scale consisting of a total of 40 points that assesses the individual in more depth, but consequently requires more time for its application [6].

The Aachen Falls Prevention Scale was developed in order to allow the elderly to perform a self-assessment of their risk of falling [19]. The Aachen Fall Prevention Scale is an easy-to-understand tool that investigates various factors that contribute to the genesis of falls and introduces a quick and safe test; it allows the individual to perform a self-assessment and increase the degree of self-perception. The authors have also created an evaluation index, the “Aachen Mobility and Balance Index” to measure the physiological risk of falling in the elderly at home; this includes the execution of performances characterized by the progressive increase of difficulty in the components of balance, mobility and grip strength required for the completion of the test. The index demonstrated a strong correlation with the Tinetti POMA Scale and a good degree of discrimination between individuals at risk of falling and not, but the time required to execute the performances and the necessary equipment make it more complex.

The Fullerton Advanced Balance (FAB) Scale is a multidimensional tool for assessing balance developed for functionally independent seniors. It aims to identify highly active seniors who are at increased risk of suffering fall-related injuries due to sensory impairments [24]. The validity of its contents is based on a theoretical analysis of the components of the static and dynamic balance, the reception and integration of the sensory components and the anticipatory and reactive postural control. Berg Balance Scale (BBS) was developed for the evaluation of both static and dynamic balance capacity. It provides a detailed balance assessment and has been extensively tested in various contexts: United States, Canada, Brazil, Australia, China, Japan, Korea and United Kingdom. It has shown accuracy in predicting falls in different types of population (elderly living in communities or suffering from chronic diseases or with intellectual and visual disabilities) and a greater sensitivity was highlighted when applied in populations affected by diseases that affect the balance (e.g., neuromuscular pathologies) both in clinical and home settings [53]. Compared to the FAB scale, it has a “ceiling effect” that does not allow it to be administered to physically active elderly people [54]. It also requires a much longer application time, about 20 min.

The Balance Evaluation Systems Test (BESTest) aims to identify the disordered systems underlying postural control responsible for poor functional balance in adults. It is widely used to evaluate six balance control systems; however, it is difficult to apply in clinical situations due to the long administration time (approximately 20–30 min). The BESTest has two shorter versions: the Mini-BESTest [18], developed in 2010, allows the evaluation of four balance control systems (compared to the six total of the original) and has been used with different clinical populations and has shown particular psychometric properties in individuals with Parkinson’s syndrome [55]. The test, however, requires a moderate amount of equipment to be completed and, moreover, it focuses on dynamic balance, without evaluating all the stability control systems; while the Brief-BESTest, developed in 2012, is a reduced version, consisting of eight items that allow an analysis of all six balance control systems evaluated by the BESTest; moreover, it requires even less time and material to complete it [56]. The comparison of the two tests shows that the Brief-BESTest is recommended as a guide for planning interventions, while the Mini-BESTest is more appropriate as a screening tool for the dynamic balance of patients [55].

The 5 Times-Sit to Stand Test (5T-STS) was developed and validated in America to establish the ability of individuals with balance disorders to perform transitional movements. A simple and quick test provided an objective measurement of the level of balance and coordination of individuals subject to various health alterations such as Sdr. Parkinson’s disease or chronic stroke [56]. Subsequently, the restriction of use of the 5T-STS test defined as unable to evaluate a population of individuals with different degrees of motor ability was criticized, which is essential if a tool is to be applied within institutionalized geriatric contexts. Similar to 5T-STS is the Dynamic Gait Index (DGI), that verifies the participant’s ability to maintain the equilibrium of walking by responding to different requests. Some studies demonstrated the high reliability of the assessment in elderly people, subjects suffering from vestibular dysfunction, multiple sclerosis and post-stroke [15]. It can be performed in both hospital and home settings, as it does not require any special equipment. It evaluates all aspects of gait, but takes a long time to administer (15 min) [15].

The Timed Up and Go (TUG) test is simple and effective, and seeks functional mobility: it provides a quick assessment of the individual’s strength, mobility capacity and dynamic balance [11]. Numerous studies have attested its validity by applying it in different contexts such as wards for acute patients or community residents [57], or to individuals with different health alterations such as Parkinson’s syndrome [58] or mental disabilities [59]. It is a simple test to implement, therefore applicable in clinical situations where the time available to the healthcare professional is a fundamental resource. Compared to the 5T-STS and the DGI, it presents multiple variables that can alter the result of the test (such as the support foot while getting up from the chair, the moment in which the stopwatch starts, the clear understanding of the tasks to be performed by the individual), has also shown reduced efficacy when administered to people with high or normal functional mobility [45]. Finally, the different cut-off values must be considered based on the age, context and pathological condition of the population being tested.

The Downton Fall Risk Index (DFRI) detects the risk of falling. The tool has been used in several studies with different purposes: to evaluate the effectiveness of a program for the elderly in post-stroke rehabilitation aimed at muscle strengthening, or to discriminate in a population of elderly who had experienced previous episodes of falls. The Tool has been extensively analyzed in contexts that do not include acute patients such as nursing homes, while when applied to the hospital environment, its ability to predict falls significantly decreases [60].

The Conley Scale assesses the risk of falls. The scale has been inserted and used in various Italian hospital contexts (mainly medicine and surgery). It is quick to use (about 2 min) and is easily understood; it is usually administered upon admission of the patient to the ward. However, the inability to identify patients not at risk of falling due to the low specificity value was contested [61]; therefore, it is recommended to use it as a preliminary evaluation to identify subjects who need a more in-depth clinical evaluation [62].

The Minimal Chair Height Standing Ability Test (MCHSAT) involves measurements of the minimum height of the chair from which the person is able to stand up. The tool has been used in several studies with different purposes: to evaluate the effectiveness of a program for the elderly in post-stroke rehabilitation aimed at muscle strengthening, or to discriminate in a population of elderly people who had experienced previous episodes of falls. The test showed, if administered in a standardized way, a high sensitivity (75%), which, combined with its simplicity of execution and speed of administration, makes it an appropriate tool for screening the risk of falling in healthcare settings [63]. From the comparison performed between the MCHSAT test and the 5T-STS, it emerged that both are less effective if performed by patients with heart disease or stroke, while in patients suffering from arthritis of the lower limbs, the 5T-STS is more effective [63].

Stopping Elderly Accidents, Deaths, and Injuries (STEADI) was created to help health professionals integrate fall risk assessment into routine practice. The tool also includes a list of fall prevention interventions for clinical use. The STEADI algorithm provides valid, unique information on the risk of falling, independently of traditional indicators based on a low level of physical health [28].

Morse Fall Scale (MFS) is a quick and easy way to assess the likelihood of a patient falling. It was developed in acute patient, rehabilitation and nursing home departments. The scale was computer tested in a simulated population and was subsequently applied to the populations of the previously mentioned departments. The Morse scale takes a few minutes to complete (approximately 2 min), allows for three different risk categories (low, medium and high) and is used in hospital settings [64]. This tool, however, does not investigate factors relevant to the risk of falls such as sensory deficits and the intake of certain drugs that can affect the mechanisms involved in the genesis of falls. For this reason, some studies have implemented a more accurate stratification of the areas investigated by the items.

A scale concerning the presence of drugs is the medication fall risk score (RxFS). It was developed as part of the pharmacist-coordinated falls prevention program in America to compensate for the lack of various drug therapy risk assessment tools [65]. The US Agency for Healthcare Research and Quality (AHRQ) has approved and recommended its use in conjunction with various nurse-administered rating scales, such as MFS and STRATIFY. The retrospective cohort study by Yazdani and Hall evaluated the efficacy of the association of RxFS with the MFS scale [26].

Falls Risk for Older People-Community Setting Screening Tool (FRHOP Com Screen) was developed by a multidisciplinary team of experts and tested in the wards of subacute patients. The Thai version of the scale has demonstrated satisfactory reliability and validity in hospitalized elderly subjects. Sensitivity and specificity are, respectively, 57% and 68%. By applying modifications to the FRHOP, the Western Health Fall Risk Assessment (WHeFRA) scale was developed by Walsh et al. and demonstrated efficacy comparable to the STRATIFY scale [20]. Austin Health Falls Risk Screening Tool (AHFRST) was developed for acute or subacute patients. This tool is administered to all patients upon entering the ward for a preliminary identification of those at risk of falling; it is fast and easy to use [29]. Comparison with the TNH-STRATIFY scale performed in the original study showed that both are unable to identify individuals not at risk of falling. Therefore, they demonstrate a low degree of “predictivity” if applied in this type of population [29].

The main elements that distinguish the assessment tools shown in the review are: the risk factors investigated (intrinsic and/or extrinsic), the sample subjected to analysis (hospital population and/or resident at home) and the way the test is used (questions to be answered and/or physical actions to be performed). Between the instruments presented between the discussion and Table 2, eight involve the execution of one or multiple physical actions, with the aim of evaluating the mechanisms that regulate the maintenance of balance and coordination in the individual (“Performance”); 26 investigate different risk factors through questions directed to the patient or elements on which the attention of the professional using the instrument is guided (“Self-report”), and finally, three scales include both the investigation of some risk factors through questionnaire, and the execution of one or more physical activities. The RxFS is excluded from this distribution, as it guides the attribution of a score exclusively based on the type of drugs taken by the individual. The risk factors investigated by the “Self-report” tools are mainly intrinsic, which means referred to the person; among these, there are: the identification of previous episodes of falls (present in 23 scales); the evaluation of the cognitive state aimed at identifying confusion, disorientation, agitation, impulsiveness or mnemonic difficulties (present in 18 scales), alterations of organs or systems and intake of drugs linked or not to specific pathological conditions, and other intrinsic variables such as age and gender. Regarding extrinsic factors, only 5 tools evaluate them in a specific way, paying attention to the context in which the individual lives. Instead, the environmental factor represented by walking aid devices is investigated by almost all the instruments presented.

Each scale, before being applied in a specific clinical context or on a population other than that for which it was created, must be properly tested, to verify its effectiveness and reliability [61]; in fact, the same instrument can have variable values of sensitivity and specificity if used in different contexts or with different populations. For example, the combined application of a test with high and stable sensitivity (e.g., TUG test) and one with a high and stable specificity (e.g., BBS) allows increasing the diagnostic precision in predicting the risk of falling. The use of a scale that evaluates the state of illness or the alterations individual (e.g., STRATIFY or HIIFRM) simultaneously with a test that evaluates the physical ability to maintain balance (e.g., BBS) can permit greater accuracy in identifying those at risk, even in different contexts [53].

## 5. Limitations

This revision has the following limitations: only the articles of the last 10 years were analyzed, the entire scenario of fall risk assessment tools present in the literature, there is not sufficient information available on the statistical characteristics of the different instruments to compare the tools or their parameters.

## 6. Conclusions

This study presents the entire scenario of fall risk assessment tools present in the literature. Between most of the tools analyzed, 23 tools target hospitalized patients, eight are used for risk assessment in home residents, while seven are applicable to both populations. The primary purpose of using a fall risk assessment tool is not to reduce falls, but to identify individuals at high and low risk [53]. In this way, the subjects that need a more in-depth analysis are identified, and the healthcare professional’s attention is focused on the main risk factors responsible of falls. To these subjects should be offered a multifactorial falls risk assessment [6].

According to some studies, to maximize the predictability characteristics of each instrument, it would be recommended to use two tests in combination.

Indeed, due to the multidimensional nature of the risk of falling, there is no “ideal” a single tool that can be used in any context or that performs a perfect risk assessment. A simultaneous application of several instruments is recommended and a direct and in-depth analysis by the healthcare professional is essential [6]. The results of the risk assessment should be discussed within the multi-professional team in order to identify the most effective interventions for preventing falls, especially among people with cognitive disorders, where the risk of fall is very common and have different etiologies [66].

## Figures and Tables

**Table 2:** Specific tools for given contests/population.

Scale, Reference, Country	Rate	Language—Year of Validation	Sample	Time of Administration	How to Use
Baptist Health High Risk Falls Assessment (BHHRFA) [30] USA.	Score items + Nurse’s clinical judgment (0–10). ≥13 a Risk of falling.	English 2014.	Hospital setting.Psychiatric field.	3 min or less.	Self-report.
WSFRAT (Wilson-Sims Fall Risk Assessment Tool) [31] USA.	0–6 Low risk; ≥7 High risk.	English 2014 and 2016.	Hospital setting.Psychiatric field.	N/a.	Self-report.
4-item Little Schmidy Pediatric Hospital Fall Risk Assessment Index [32] USA.	Score 0–4. ≥1 a Risk of falling ≥3 High risk.	English 2016.	Hospital setting.Pediatric field.	N/a.	Self-report.
Humpty Dumpty Fall Scale (HDS) [33] USA.	Score 0–23. 7–11 Low risk; 12–23 High risk.	English 2007.	Hospital setting.Pediatric field.	N/a.	Self-report.
Bayındır Hospital Risk Evaluation Scale for In-hospital Falls of Newborn Infants [34] Turkey.	1–3 Low risk. ≥4 High risk.	Turkish 2010.	Hospital setting.Pediatric field.	N/a.	Self-report.
KINDER 1 Fall Risk Assessment Tool [35] USA.	Answer “Yes” to any item = High risk of falling.	English 2013.	Hospital setting.Emergency department.	N/a.	Self-report.
Memorial Emergency Department (MED-FRAT) [36] USA.	Score 0–14. 1–2 Low risk; 3–4 Moderate; ≥5 High risk.	English 2013.	Hospital setting.Emergency department.	N/a.	Self-report.
Casa Colina Fall Risk Assessment Scale (CCFRAS) [37] USA.	Score 0–260. If you answer “Yes” to the item “Tetraplegia” = Low risk. ≥80 High risk.	English 2014.	Hospital setting.Rehabilitation field.	N/a.	Self-report.
Predict_FIRST [38] Australia.	Score 0–5. Probability of falling based on the score: 0 = 2%; 1 = 4%; 2 = 9%; 3 = 18%; 4 = 33%; 5 = 52%.	English 2010.	Hospital setting.Rehabilitation field.	N/a.	Self-report.
Marianjoy Fall Risk Assessment Tool [39] USA.	Score 0–10 ≥4 a Risk of falling.	English 2005.	Hospital setting.Rehabilitation field.	N/a.	Self-report.
Simple clinical scale [40] France.	Score 0–16. 0–4 Low risk; 5–10 Moderate risk; 11–16 Other risk.	French 2010.	Screening of population. Home care.	N/a.	Self-report.
Home Falls and Accidents Screening Tool (HOME FAST) [41] Australia.	Score 0–25. A higher score = herefore a higher risk of falling.	English 2002.	Screening of population. Home care.	N/a.	Self-report.
The Stroke Assessment of Fall Risk (SAFR) [42] USA.	Score 0–49 0 = Low risk; 49 = Higher risk.	English 2011.	Hospital setting.Stroke patients.	N/a.	Self-report.
The Royal Melbourne Hospital Falls Risk Assessment Tool (RMH FRAT) [43] Australia.	0–4 Low risk; 5–14 Medium risk; ≥15 High risk.	English 1997.	Hospital setting.Stroke patients.	N/a.	Self-report.
Sydney Falls Risk Screening Tool [44] Australia.	Score ≥ 33 = a Risk of falling.	English 2018.	Hospital setting.Stroke patients.	N/a.	Self-report.
Outdoor Falls Questionnaire [45] USA.	A higher score indicates a higher risk of falling.	English 2015.	Screening of population. Stroke patients.	20 to 25 min.	Self-report.
Questionnaire for Fall Risk Assessment in the Elderly [46] Brazil.	N/a.	Brazilian 2017.	Screening of population. Stroke patients.	N/a.	Self-report.
